# Frequency of re-entry injury in repeat cardiac surgery and associated risk factor evaluated at a newly established cardiac center

**DOI:** 10.12669/pjms.41.11.12208

**Published:** 2025-11

**Authors:** Muhammad Asif Shams, Hira Hameed, Danial Khattak, Abdul Nasir

**Affiliations:** 1 Muhammad Asif Shams, Peshawar Institute of Cardiology, Peshawar, Pakistan; 2 Hira Hameed, Peshawar Institute of Cardiology, Peshawar, Pakistan; 3 Danial Khattak, Peshawar Institute of Cardiology, Peshawar, Pakistan; 4 Abdul Nasir, Peshawar Institute of Cardiology, Peshawar, Pakistan

**Keywords:** 30-day mortality, Cardiopulmonary bypass, Central cannulation, Postoperative complications, Repeat cardiac surgery, Re-entry injury

## Abstract

**Background & Objective:**

Repeat cardiac surgery is often complicated by re-entry injuries and other risks, especially in patients with a history of prior cardiac procedures. The associated reason of repeat surgeries include complications such as graft failure, valve dysfunction, or disease recurrence. The procedure is categorized as higher risk profiles compared to primary surgical intervention. This study aimed to assess the frequency of re-entry injuries and other early complications in patients undergoing repeat cardiac surgery at a newly established cardiac center.

**Methodology:**

The study included patient’s data who visited cardiac surgery department of Peshawar Institute of Cardiology between January 2021 to February 2025. This retrospective study included 28 patients who underwent repeat cardiac surgery. Data on demographics, pre-operative status, comorbidities, and surgical procedures were collected from the hospital’s management information system (HMIS) and electronic medical records (EMR). The primary outcomes were re-entry injury frequency, postoperative complications, and 30-day mortality.

**Results:**

The study included 64.3% female patients, with a mean age of 38.89 years. Common pre-operative conditions included hypertension (32.1%) and advanced heart failure, with 71.4% of patients classified as NYHA class III and IV. The most frequent procedures were redo mitral valve replacement (MVR) and pseudoaneurysm repair. Central cannulation was used in 78.57% of cases, with a mean bypass time of 140.23 minutes.

**Conclusion:**

This study provides important insights into the characteristics, procedures, and outcomes of patients undergoing repeat cardiac surgery at a newly established cardiac centre with no re-entry injury. The findings indicate that the majority of patients were female, mostly presenting advanced heart failure and requiring complex, multi-valve procedures. Central cannulation was the predominant method for cardiopulmonary bypass.

## INTRODUCTION

Repeat cardiac surgery is becoming increasingly common as the number of patients surviving initial cardiac interventions, such as coronary artery bypass grafting (CABG) or valve repair/replacement, continues to rise.^1^ Over time, many of these patients may require re-intervention due to graft failure, disease progression, or valve dysfunction. While repeat surgeries can provide a critical life-saving opportunity for these patients, they are not without risks, with one of the most significant complications being re-entry injury.^2^

Re-entry injury refers to the damage that occurs during the reopening of previously accessed cardiac structures, such as the sternum, coronary vessels, or valve annuli. This can result in injury to adjacent tissues, excessive bleeding, or compromised grafts, ultimately leading to poor clinical outcomes.^3^ The risk of such injuries is particularly high due to factors like scar tissue formation, adhesions, and anatomical changes resulting from prior surgical procedures.^4^ Despite the known challenges, the incidence of re-entry injury remains a critical concern, especially in settings where surgical teams may be less experienced with repeat procedures.^5^

In newly established cardiac centers, where teams may be in the process of gaining proficiency in handling complex cases, the frequency and severity of re-entry injuries in repeat cardiac surgeries may differ from those seen in more established institutions.^6^ Limited research has specifically focused on this setting, with only a few studies examining the frequency and impact of re-entry injuries in newly operational cardiac patients.^7^ Given the importance of patient safety and surgical outcomes in such institutions, it is crucial to explore how the frequency of re-entry injury might vary and how it can be minimized through enhanced surgical planning, technique refinement, and patient management strategies.^8^

The current study aimed to evaluate the frequency of re-entry injuries in repeat cardiac surgery patients at a newly established cardiac center. By analyzing surgical outcomes and identifying risk factors associated with these injuries, the research seeks to provide insight into the frequency and potential mitigation strategies for re-entry injuries, contributing to improved patient care in this clinical context.

## METHODOLOGY

This retrospective observational study was conducted over a period from January 2021 to February 2025 at cardiac surgery department of Peshawar Institute of Cardiology. A total of n = (28) patients were enrolled. This study included consecutive patients aged 18 years or older who had undergone repeat cardiac surgery at a newly established cardiac center, regardless of whether cardiopulmonary bypass was used. It included elective, urgent, emergent, and salvage cases. Patients with incomplete medical records were excluded. Data on baseline patient characteristics, details from previous cardiac surgeries, peri-operative information, and postoperative outcomes were retrospectively obtained from the hospital’s management information system (HMIS) and electronic medical records (EMR).

### Ethical Approval:

The study was approved by the Institutional Review Board (IRB) of the Peshawar Institute of Cardiology (IRC/25/175; dated: March 19, 2025).

The primary endpoints of the study were the frequency of re-entry injury during repeat surgery, postoperative complications, and 30-day mortality. During surgery, the sternal wires were cut interiorly and bent back but were not removed to protect the underlying structures. An oscillating saw was used to divide the sternum. After the posterior table of the sternum was divided, the wires were removed, and sharp dissection was performed with scissors to separate the sternum from the underlying structures. The dissection plane closely followed the sternum, along the diaphragmatic surface, extending upward toward the right atrium and aorta. Dissection around the aorta was done cautiously to avoid penetration or extension beneath the adventitia. Cardiopulmonary bypass (CPB) was initiated for the dissection of the left heart, with mild systemic hypothermia (30-32°C). Myocardial protection was achieved using antegrade blood cardioplegia during induction and continuous retrograde blood cardioplegia during maintenance.

### Statistical Analysis:

Data from the study were analyzed using SPSS (Statistical Program for Social Science) version 25. Continuous variables were presented as mean ± standard deviation, while categorical variables were reported as frequencies and percentages.

## RESULTS

The Table-I presents a breakdown of key characteristics for a sample group undergoing repeat cardiac surgery. Among the participants, 64.3% were female (n=18) and 35.7% were male (n=10). Regarding comorbidities, 32.1% of the patients (n=9) had hypertension. The majority of patients were classified according to the Canadian Cardiovascular Society (CCS) classification, with 7.1% (n=2) in CCS I, 42.9% (n=12) in CCS II, and 50% (n=14) in CCS III, indicating a predominantly moderate to severe degree of angina. When classified according to the New York Heart Association (NYHA) functional classification, 7.1% (n=2) were in NYHA II, 71.4% (n=20) in NYHA III, and 21.4% (n=6) in NYHA IV, showing that most patients had a severe reduction in functional capacity due to heart failure. The mean age of the participants was 38.89 years (±13.73), and the mean body mass index (BMI) was 24.10 (±4.68), suggesting a generally healthy weight range among the sample group. These characteristics provide important context for understanding the clinical status of the patients and potential factors that may influence surgical outcomes.

The Table-II outlines the various procedures performed on patients undergoing repeat cardiac surgeries. Among the procedures, the most frequent was redo mitral valve replacement (MVR), which occurred in 14.3% of cases (n=4). Other common procedures included pseudoaneurysm repair, which was performed in 14.3% (n=4) of cases, and redo tricuspid valve repair (TVR), which accounted for 7.1% (n=2) of the procedures. Other specific combinations of surgeries were also performed, such as redo MVR with aortic valve repair (n=1, 3.6%), redo aortic valve replacement (AVR) with aortic root enlargement and tricuspid valve repair (n=1, 3.6%), and several other variations of valve repairs and replacements. A variety of other combinations were conducted with individual frequencies of 3.6%, including procedures like redo MVR with AVR, redo pulmonary valve replacement, and redo ruptured sinus of Valsalva repair. Additionally, salvage AVR was performed in 3.6% (n=1) of patients, indicating a more complex or high-risk situation. These results highlight the diverse nature of the repeat procedures conducted in this cohort, with valve-related interventions being the most frequent, reflecting the complexity and variability of the cardiac conditions being treated.

**Table-I:** Demographic profile and pre-operative assessment of patients: (n=28).

Characteristics	Frequency (n)	Percentages (%)
Male	10	35.7
Female	18	64.3
Hypertension	09	32.1
CCS I	02	7.1
CCS II	12	42.9
CCS III	14	50
NYHA II	02	7.1
NYHA III	20	71.4
NYHA IV	06	21.4
Age (years) (Mean±SD)	38.89 ±13.73	
BMI (Mean±SD)	24.10±4.68	

**Table-II:** Procedures of Re-do Cardiac surgeries: (n=28).

Procedures	Frequency (n)	Percentages (%)
MVR+TVR	1	3.6
Pseudoaneurysm repair	4	14.3
Redo MVR+aortic valve repair	1	3.6
Redo MVR+AVR+TV repair	1	3.6
Redo AVR+aortic root enlargement +TV repair	1	3.6
Redo AVR+TVR	1	3.6
Redo MVR, AVR, TV repair	1	3.6
Redo MVR+TVR	1	3.6
Redo TVR	2	7.1
Redo AVR	1	3.6
Redo AVR+ Fistula repair	1	3.6
Redo AVR+MVR	1	3.6
Redo CABG	1	3.6
Redo DVR	1	3.6
Redo MVR	4	14.3
Redo MVR+ Aortic valve repair	1	3.6
Redo MVR+AVR	1	3.6
Redo Pulmonary valve replacement	1	3.6
Redo ruptured sinus of Valsalva repair	1	3.6
Redo-MVR+AVR+ repair	1	3.6
Salvage AVR	1	3.6

Table-III presents the early comorbidities and intra-operative measurements of patients undergoing repeat cardiac surgeries. Among these patients, 10.7% (n=3) required re-exploration during the postoperative period, while 14.28% (n=4) experienced re-intubation and 14.28% (n=4) had to be re-admitted, indicating significant complications requiring further intervention after surgery. Additionally, 3.6% (n=1) of patients suffered a stroke during the perioperative period, and 3.6% (n=1) experienced 30-day mortality, highlighting the risks associated with repeat cardiac surgeries. The mean cross-clamp time was 195.51 minutes (±73.9), reflecting the duration for which the aorta was occluded during surgery, while the mean bypass time was 140.23 minutes (±74.8), representing the total time the patient was on cardiopulmonary bypass. These measurements are important indicators of the complexity and duration of the surgical procedure, as longer cross-clamp and bypass times are generally associated with higher risks of complications. Overall, the data underscores the challenging nature of repeat cardiac surgeries, with a notable proportion of patients experiencing significant early complications.

**Table-III:** Early Comorbidities and intra-operative measurement: (n=28).

Characteristics	Frequency (n)	Percentages (%)
Re-Exploration	3	10.7
Re-Intubation	4	14.28
Re-Admission	4	14.28
Stroke	1	3.6
30 days mortality	1	3.6
Cross clamp time (minutes)(Mean±SD)	195.51±73.9	
By pass time (minutes) (Mean±SD)	140.23±74.8	

**Fig.1:**
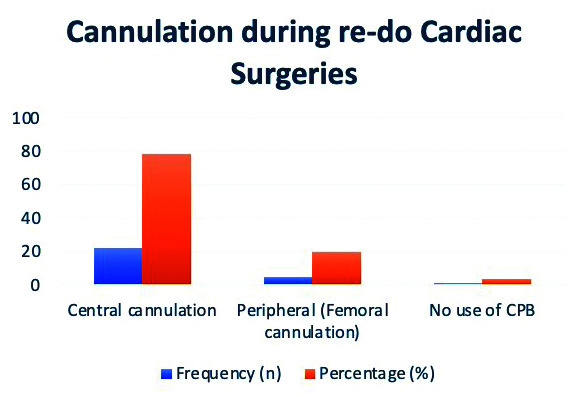
Cannulation during re-do cardiac surgeries: (n=28).

As presented by Fig.1, the most commonly employed method was central cannulation, which was utilized in 78.57% (n=22) of the cases, reflecting its widespread use in cardiac surgeries. Central cannulation involves the insertion of a cannula into the heart or aorta directly, typically through the ascending aorta or right atrium, and is often preferred for its reliability in providing adequate blood flow during the procedure. The next most frequent technique was peripheral femoral cannulation, used in 20% (n=5) of the cases. This approach involves cannulating the femoral artery and vein to support cardiopulmonary bypass, often employed when central access is not feasible or in specific types of surgery, such as those requiring minimal invasiveness. A smaller proportion of patients, 3.57% (n=1), the heart was operated on without the use of cardiopulmonary bypass. This technique is increasingly used to reduce the risk of complications associated with bypass procedures. Overall, central cannulation was the predominant choice, though the data also highlights the use of peripheral cannulation and OPCAB as alternative approaches based on patient-specific factors or surgical considerations.

## DISCUSSION

The findings of this study provide valuable insight into the characteristics and outcomes of patients undergoing repeat cardiac surgery in a newly established cardiac center. In this cohort study, the majority of patients were female (64.3%), which aligns with previous studies reporting a higher prevalence of heart disease in women, particularly in later stages of life. The sample population was characterized by a relatively young mean age (38.89 years), which is consistent with other research suggesting that younger patients are more likely to undergo repeat surgeries due to the long-term survival after the initial cardiac intervention.^9^ Furthermore, a significant proportion of patients had severe functional limitations, with most classified under NYHA III and IV, indicating the advanced stage of heart failure in this population, which corresponds with findings from earlier studies highlighting the higher incidence of repeat surgeries in patients with advanced heart failure.^10^

Regarding the procedures performed, the most common interventions were redo mitral valve replacements (14.3%) and pseudoaneurysm repairs (14.3%), both of which are in line with the frequent need for valve-related interventions in patients requiring re-operation. The data also show a wide range of procedural combinations, reflecting the complexity of the cases in this cohort. These results are consistent with other studies indicating that repeat cardiac surgeries often involve multi-valve procedures or a combination of repairs. In terms of intraoperative complications, the incidence of re-exploration (10.7%) and re-intubation (14.28%) are consistent with previous studies that report increased complication rates in patients undergoing repeat surgeries due to the technical difficulties posed by scar tissue, adhesions, and anatomical changes.^11^ Additionally, the 30-day mortality rate of 3.6% observed in this study is comparable to other reports that suggest repeat cardiac surgeries carry a higher risk of early mortality compared to primary surgeries.^12^

Regarding cannulation techniques, central cannulation was the most commonly employed method (78.5%), which is consistent with previous reports that favor central cannulation for its reliability and ease in repeat surgeries.^13^,^14^ However, peripheral femoral cannulation (20%) and no use of CPB (3.57%) were also used, reflecting the individual surgical considerations and the evolving use of less invasive techniques to reduce complications such as stroke or organ dysfunction.^15^,^16^

Overall, this study contributes to the growing body of knowledge on repeat cardiac surgeries, particularly in newly established cardiac centers, and highlights both the challenges and outcomes associated with these complex procedures. These findings are consistent with previous studies but also underline the unique challenges faced by newly established centers, including a potentially higher rate of complications due to the learning curve and limited resources. Moreover, it’s imperative to state that none of our patient faced any kind of re-entry injuries. This is due to the fact that we had adopted safe pre-operative optimization techniques, like doing CT scan in order to ensure the safe distance of heart and sternum. Moreover, we also adopted safe surgical techniques by using sternum clips, silk suture to lift the sternum. Further studies in these settings are needed to optimize surgical strategies and improve patient outcomes.

## CONCLUSION

This study provides important insights into the characteristics, procedures, and outcomes of patients undergoing repeat cardiac surgery at a newly established cardiac center. The findings indicate that the majority of patients were female, mostly presenting with advanced heart failure and requiring complex, multi-valve procedures. The predominant use of central cannulation reflects the standard approach in repeat surgeries, although peripheral femoral cannulation and no use of CPB were also utilized depending on individual case requirements. These results align with those of previous studies, reinforcing the higher complexity and risks associated with repeat cardiac surgeries. Overall, the study underscores the importance of refining surgical techniques and strategies to reduce complications, particularly in newly established cardiac centers where experience and resources may be limited. Further research is necessary to optimize outcomes and improve patient safety in these settings.

### Author’s Contribution:

**MAS:** Performed data analysis and drafted the manuscript.

**HH and DK:** Did the statistical analysis, **AN:** supervised the study, revised the manuscript.

**MAS and AN:** Principle & corresponding authors respectively, are responsible for data validity.

All authors were involved in editing the manuscript. All authors have read and approved the final version of manuscript and are accountable for the integrity of the study.
